# Sensing, Imaging, and Computation as One: Rethinking Microfluidic Platform Design

**DOI:** 10.3390/mi17070807

**Published:** 2026-07-01

**Authors:** Sevketcan Sarikaya, Horacio D. Espinosa

**Affiliations:** 1 Department of Mechanical Engineering, Northwestern University, Evanston, IL 60208, USA; 2 Chan Zuckerberg Biohub Chicago, Chicago, IL 60642, USA

## 1. The Measurement Problem: Biology Is Dynamic; Our Tools Are Not

Living systems do not operate in snapshots. Cells secrete signaling molecules in transient bursts, tissues remodel their mechanical landscapes over hours, and organoids undergo morphological transitions that reflect shifting fate decisions within their architectures. These processes are continuous, spatially heterogeneous, and inherently multimodal. They span molecular, mechanical, and morphological domains simultaneously and unfold across timescales ranging from minutes to days.

Yet the dominant analytical paradigm in cell and tissue biology remains anchored to endpoint assays. Supernatants are collected at fixed intervals and analyzed via ELISA. Cells are fixed, stained, and imaged at terminal time points. Genomic and proteomic profiles are extracted from lysed populations. Each of these approaches collapses a rich, dynamic, and spatially structured process into a single static measurement: one number, one image, and one omics snapshot, averaged across an entire population. The resulting data describe what a system looked like at the moment it was destroyed.

This mismatch between the temporal and spatial richness of biology and the limitations of conventional measurement is not primarily a biological problem. It is an engineering problem, one that microfluidic and biosensing microsystems are well positioned to solve.

Several converging developments make this argument particularly timely. Three-dimensional tissue models, including organoids, microphysiological systems, and organ-on-chip devices, have matured to the point where they faithfully recapitulate key aspects of human organ physiology [1,2,3]. Regulatory agencies have shown growing interest in organ-on-chip and other new methodologies, with the U.S. FDA actively engaging in implementing evaluation frameworks for microphysiological systems as alternatives to animal models [3,4]. The CEN/CENELEC Focus Group on Organ-on-Chip published its first international standardization roadmap in 2024, signaling that the field is moving from the proof-of-concept stage to reproducible, translatable practice. At the same time, advances in microfabricated sensor technologies, label-free optical detection, and microfluidic integration have made embedded, real-time biosensing increasingly feasible [5,6,7]. The technical foundations are in place. What remains missing is the design philosophy with which to assemble them into coherent analytical platforms.

## 2. Microfluidic Platforms as Analytical Instruments

Over the past two decades, organ-on-chip and microfluidic culture platforms have made remarkable progress in recapitulating tissue-level architecture. Devices now reproduce barrier functions, vascular perfusion, mechanical strain, and multi-organ crosstalk with increasing physiological fidelity [1,3,8]. These achievements have been widely celebrated, and rightly so. However, a critical asymmetry persists: most microfluidic systems are designed primarily as culture environments, with analytical readouts performed off-chip using the same conventional assays—including ELISA, Western blot, qPCR, and end-point microscopy—that the field originally set out to improve upon. As Zhu et al. noted in their review of integrated biosensors for organ-on-chip applications, characterization of these sophisticated tissue constructs still relies overwhelmingly on conventional, labor-intensive approaches [5].

This represents a missed opportunity. The same physical features that make microfluidics powerful for cell culture, including low reagent volumes, laminar flow, controlled mass transport, and precise geometric definition, also make them ideally suited for embedded sensing, analyte pre-concentration, and real-time readouts. A 10 µL chamber that sustains a tissue construct can simultaneously serve as a detection volume in which secreted biomarkers accumulate to measurable concentrations far more rapidly than in bulk culture. Integrated electrodes, optical waveguides, or plasmonic nanostructures can transduce molecular binding events without removing the sample from its biological context [6,7,9].

**Figure 1:**
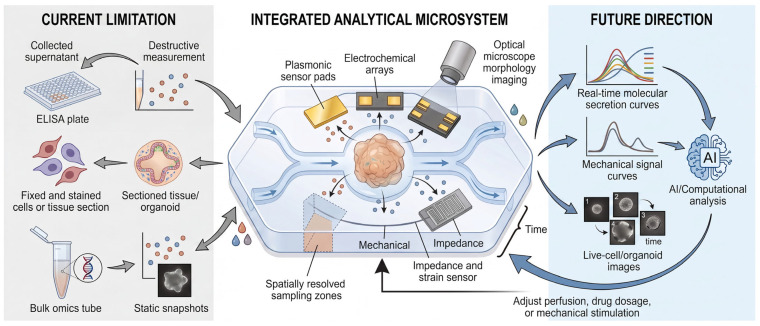
Three views of microfluidic and biosensing microsystems for cell and tissue analysis. Current limitation: Conventional workflows rely on destructive endpoint assays, including ELISA applied to collected supernatants, fixed and stained tissue sections, and bulk omics from lysed populations, each of which produces a static snapshot averaged across the sample. Integrated analytical microsystem: The platform itself measures the biology it supports, with embedded plasmonic biosensors, electrochemical arrays, optical microscopy for morphology, and impedance and strain sensors all coupled to spatially resolved sampling zones around a three-dimensional tissue construct. Future direction: Continuous multimodal data streams, including real-time molecular secretion curves, mechanical signal traces, and live-cell or organoid imaging, feed AI-driven computational analysis that closes the loop by adjusting perfusion, drug dosage, or mechanical stimulation in real time.

What is needed is a shift in design philosophy from “microfluidic device plus external assay” to “microsystem as integrated analytical instrument.” The platform itself should measure, not merely house, the biology it supports. This shift is illustrated in Figure 1, which contrasts conventional endpoint workflows with an integrated multimodal microsystem and the closed-loop, computationally augmented platforms toward which the field is moving. It has been demonstrated that integrated electrochemical immunosensors; physical sensors for pH, temperature, and dissolved oxygen; and optical readouts within a multi-organ-on-chip platform enable automated, continual, and in situ monitoring of organoid responses to pharmaceutical compounds [10]. Similarly, Misun et al. developed a multi-analyte biosensor interface for real-time monitoring of 3D microtissue spheroids, showing that glucose and lactate dynamics could be tracked continuously in hanging-drop networks [11]. An additional example is the live-cell analysis device (LCAD), a microwell-array microfluidic platform that uses localized electroporation not only for intracellular delivery but also for non-destructive sampling of cytosolic contents from live cells [12]. By directly interfacing the LCAD with self-assembled monolayers for MALDI mass spectrometry (SAMDI), Patino et al. quantified protein tyrosine phosphatase (PTP) activity sampled from human MDA-MB-231 breast cancer cells. This is biologically significant because PTPs, together with protein kinases, regulate signaling pathways that control cell growth, survival, and proliferation; their dysregulation is closely associated with abnormal cancer-cell proliferation. Thus, LCAD-SAMDI illustrates how an integrated microsystem can move beyond extracellular biomarkers and interrogate intracellular enzymatic activity in live cells, opening a path toward longitudinal studies of drug response, including evaluation of PTP inhibitors, without destroying the biological sample.

A related advance is the multi-well localized electroporation device (LEPD), which integrates cell manipulation with automated microscopy and machine-learning-based image analysis [13]. In this platform, localized electroporation enables delivery of biomolecular cargo, including siRNA, into sensitive cell types while preserving viability and allowing downstream image-based phenotyping. As an example, Patino et al. delivered siRNA targeting OCT4/POU5F1 into human induced pluripotent stem cells and used deep-learning segmentation with feature extraction to quantify changes in OCT4 expression, beta-catenin localization, and cell morphology. OCT4 knockdown reduced OCT4 protein levels and was accompanied by altered beta-catenin organization, including increased total beta-catenin signals and changes in its membrane and nuclear distribution. These observations are important because OCT4 and Wnt/beta-catenin signaling are central regulators of pluripotency, self-renewal, and differentiation. The LEPD example therefore demonstrates how microsystems can combine perturbation, imaging, and computational analysis to connect a defined molecular intervention with spatial protein reorganization and cell-state transitions.

More broadly, these examples point to a larger trend in which micro and nano technologies are no longer isolated tools for culture, delivery, or analysis but rather components of integrated cell-engineering workflows [14]. Microfluidic sorting, nano-engineered intracellular delivery, live-cell cytosolic sampling, embedded sensing, high-content imaging, and computational analysis can be combined into platforms that manipulate cells and measure their responses within the same experimental architecture. This integration is especially important for next-generation cell therapy, disease modeling, and drug screening, where the relevant biological information spans molecular activity, spatial organization, morphology, and function over time.

This trajectory is reflected in recent advances that push individual sensing modalities toward the resolution and continuity that integrated microsystems will require. For mechanical readouts, Saif et al. developed a multifunctional microfabricated sensor that performs longitudinal measurements of cell traction force, matrix stiffness, and matrix remodeling in self-assembled 3D tissues while applying mechanical stimulation [15]. For electrophysiological readouts, Rogers et al. created a soft, shape-conformal three-dimensional electronic framework that envelops a neural organoid with hundreds of microelectrodes, enabling high-resolution and non-destructive mapping [16]. For molecular readouts, Kelley et al. established active-reset electrochemical aptamer sensors capable of continuous, real-time monitoring of protein biomarkers in vivo, addressing a central obstacle to tracking secreted proteins [17]. These advances, spanning mechanical, electrical, and molecular sensing, illustrate how the individual building blocks of the integrated analytical microsystem envisioned here are rapidly maturing.

These recent demonstrations remain among the most complete examples of what a truly integrated analytical microsystem can look like. The field has already produced several technically sophisticated demonstrations of integrated sensing. The challenge is therefore not whether such integration is technically feasible (it demonstrably is) but why it has not become routine practice. The bottleneck is one of design culture and standardization, not fundamental technology.

## 3. The Need for Multimodal Measurement

No single measurement modality captures biological function. Secretome profiling reveals what cells release but is blind to mechanical state and morphology; imaging tracks shape and dynamics but not molecular identity; and omics provides molecular inventories at the cost of temporal and spatial resolution. Each modality, taken alone, offers a partial picture.

The real power of integrated microsystems lies in combining complementary readouts within the same device and the same experiment. Plasmonic or electrochemical biosensors can monitor secreted cytokines or metabolites in real time [5,6,7]. Impedance-based measurements or embedded strain sensors can track the mechanical properties of cells and extracellular matrix [18]. Live-cell microscopy, whether brightfield, fluorescence, or label-free, can capture morphological dynamics, migration patterns, and proliferation kinetics. When these modalities operate in concert, they enable something that no isolated assay can: the ability to link molecular activity to functional phenotype within the same cell population, at the same time, without sacrificing spatial context.

This is not simply a matter of collecting more data. It is a matter of collecting the right data in the right relationships. A cytokine spike that coincides with a mechanical stiffening event and a morphological transition tells a fundamentally different story than any one of those observations alone. Multimodal integration transforms measurement from description into mechanistic insight. Furthermore, because microfluidic systems can isolate and track individual cells or small clusters, they offer a route to capturing cell-to-cell variability that bulk population measurements inevitably mask, an increasingly important consideration as single-cell heterogeneity is recognized as a driver of drug resistance, differentiation bias, and disease progression.

These modalities are not equally mature. Continuous monitoring of bulk physicochemical parameters, including pH, dissolved oxygen, glucose, and lactate, is relatively well established and commercially accessible. Continuous detection of secreted proteins is considerably harder: real-time cytokine monitoring, multiplexed secretome analysis, and spatially resolved capture of low-abundance paracrine factors remain largely at the proof-of-concept stage. Recognizing this gradation in technology-readiness matters, both because it tempers claims about what integrated platforms can deliver today and because it identifies where engineering effort is most needed.

## 4. Organoids and Tissue Models Demand Better Analytical Infrastructure

The analytical gap described above is growing wider, not narrower. Three-dimensional culture models, including organoids, spheroids, and microphysiological systems, are now central tools in drug screening, disease modeling, developmental biology, and personalized medicine [2,3,19]. Their adoption reflects a well-established limitation of conventional two-dimensional culture: flat monolayers fail to capture the architecture, cell–cell interactions, and microenvironmental gradients that govern tissue-level behavior in vivo.

Yet the analytical tools applied to these increasingly sophisticated biological models remain, for the most part, adapted from two-dimensional workflows. Organoids are dissociated for flow cytometry, sectioned for immunohistochemistry, or lysed for bulk molecular profiling, each of which destroys the very three-dimensional organization that motivated their use. The geometric complexity, diffusion barriers, and cellular heterogeneity of 3D constructs amplify every limitation of endpoint assays. Gradients that exist within a 200 µm spheroid are invisible to a measurement that averages over the entire structure. Rare cell states at the periphery are lost when the population is pooled.

The convergence of organoid technology with organ-on-chip platforms, sometimes called organoids-on-a-chip, has created tissue models of unprecedented complexity [1]. Vascularized organoids; multi-organ circuits linking heart, liver, bone, and skin [20]; and patient-derived microphysiological systems now offer levels of physiological relevance that would have been difficult to imagine a decade ago. But physiological relevance demands analytical sophistication in the platform. A microphysiological system that faithfully recreates a liver–kidney axis but is interrogated only via terminal ELISA is fundamentally under-instrumented for the questions it was designed to answer.

As biological models grow more complex, analytical platforms must follow. Microsystem designers should treat integrated, spatially resolved, and non-destructive measurement not as a secondary feature but as a first-order design constraint, one that is specified alongside channel geometry, flow rates, and material selection from the earliest stages of device development.

## 5. Dynamic, Spatially Resolved, and Longitudinal Analysis as a Design Principle

If the arguments above hold, i.e., that biology is dynamic, that multimodal measurement is necessary, and that three-dimensional models amplify the inadequacy of endpoint assays, then a clear set of design principles follows for the next generation of microsystems.

First, real-time or near-real-time monitoring should become the default rather than the exception. This will require on-chip sensor architectures with sufficient sensitivity, stability, and resistance to drift for continuous operation over the timescales relevant to biological experiments (typically hours to weeks rather than minutes). Sensor recalibration strategies, whether through integrated reference channels or periodic standard injection, must be addressed at the design stage. Recent reviews have catalogued a growing repertoire of electrochemical, optical, and physical sensors that have been successfully integrated with organ-on-chip devices for continuous monitoring of pH, dissolved oxygen, metabolites, and secreted biomarkers [5,6,7,9], yet the majority of published platforms still report measurements at isolated timepoints rather than as continuous data streams.

Second, spatial resolution must be preserved. For tissues and organoids, gradients, niches, and local cell–cell interactions are not noise; they are the biology. Microsystem geometries should be designed to maintain positional information during analyte capture rather than pooling effluent into a single outlet that erases the spatial structure of the source. Multiplexed sensor arrays, zone-specific sampling channels, and imaging-compatible device architectures all contribute to this goal. Imaging-integrated microfluidic systems that combine real-time optical monitoring with fluidic control have already shown how spatial and temporal information can be preserved within a single experimental workflow [10,21].

Third, longitudinal measurement tracking the same biological unit—whether a single cell, spheroid, or tissue construct—over the full duration of an experiment should be a core capacity. Whereas real-time monitoring concerns the temporal density of data acquisition, longitudinal measurement concerns the identity and continuity of what is being measured. The two are complementary: continuous sensing reveals dynamics, while preserved sample identity allows transient signals to be correlated with downstream functional outcomes in the same biological entity. Zhang et al.’s multi-organ platform demonstrated this principle by tracking organoid drug responses non-destructively over days, revealing dose-dependent dynamics that were invisible to conventional endpoint analysis [10]. Such demonstrations should be the starting point for future platform design, not the exception.

Non-destructive, continuous, and spatially aware measurement is a prerequisite for understanding how living systems actually behave.

## 6. Looking Forward: Toward Closed-Loop, Computationally Augmented Platforms

The vision outlined here, one of integrated, multimodal, dynamic, and spatially resolved microsystems, naturally extends toward platforms that are not merely instruments of observation but active participants in experimental control. Future devices may combine embedded biosensors with on-the-fly image analysis and machine learning algorithms to enable closed-loop or semi-closed-loop workflows: systems that detect a change in secreted cytokine concentration, identify a corresponding morphological shift, and adjust perfusion rate, drug dosage, or mechanical stimulation in response, all without human intervention. Early proof-of-principle demonstrations are emerging, including adaptive microfluidic systems that use deep-learning-based sensor networks to modulate on-chip parameters in real time [22], and recent reviews have begun to map the broader integration of artificial intelligence with microfluidic and organ-on-chip platforms [23,24]. Significant barriers remain before such closed-loop operation becomes practical. These include sensor drift over experimental timescales, the data sparsity inherent in low-throughput biological experiments, device-to-device variability, and the fundamental difficulty of validating autonomous perturbation when the biological ground truth is itself uncertain. Addressing these challenges will require not only better algorithms but also the validation and standardization efforts discussed above. Such platforms would transform microsystems from passive measurement tools into adaptive experimental environments.

Realizing this vision will require progress on several fronts beyond device fabrication. Standardization of modular sensor interfaces will be essential so that biosensing elements can be combined, swapped, and scaled without redesigning the entire platform. The CEN/CENELEC Focus Group Organ-on-Chip Standardization Roadmap has identified harmonized terminology, minimum reporting requirements, and engineering standards for device design and qualification as critical priorities for the field. Similar efforts are emerging from other regulatory and standards organizations [25]. Common data formats and calibration protocols will be needed to ensure that multimodal datasets generated across different laboratories are reproducible and comparable. Additionally, the integration of computational analysis, from signal processing to AI-assisted phenotypic classification, must be treated as an intrinsic part of the platform architecture, not a post hoc software layer applied after the experiment is complete.

The opportunity before the microsystems community is significant, but it requires a deliberate shift in ambition. The goal is not simply to miniaturize existing experiments or add one more sensor to an existing chip. It is to redefine how biological function is measured: to build unified platforms in which sensing, imaging, and computation operate as a single, coherent analytical workflow. The next generation of microfluidic and biosensing systems should not only sustain living biology but interrogate it continuously, across multiple dimensions, and with the resolution that its complexity demands. The field of microsystems has the fabrication expertise, sensor technologies, and microfluidic architectures required to make this possible. What it needs now is the matching design philosophy.

## Data Availability

Not applicable.
